# Alterations in Skin Temperature and Sleep in the Fear of Harm Phenotype of Pediatric Bipolar Disorder 

**DOI:** 10.3390/jcm3030959

**Published:** 2014-08-22

**Authors:** Patricia J. Murphy, Mark G. Frei, Demitri Papolos

**Affiliations:** 1 Parallax Innovations LLC, 22 Crescent Rd., Westport, CT 06880, USA; E-Mails: patricia.jane.murphy@gmail.com (P.J.M.); markgfrei@gmail.com (M.G.F.); 2 The Juvenile Bipolar Research Foundation, 277 Martine Avenue, White Plains, NY 10601, USA

**Keywords:** pediatric bipolar disorder, Fear of Harm, phenotype, thermoregulatory deficit, distal-proximal temperature gradient, sleep disturbance, parasomnias, sleep-onset latency, sleep diary, actigraphy

## Abstract

In children diagnosed with pediatric bipolar disorder (PBD), disturbances in the quality of sleep and wakefulness are prominent. A novel phenotype of PBD called Fear of Harm (FOH) associated with separation anxiety and aggressive obsessions is associated with sleep onset insomnia, parasomnias (nightmares, night-terrors, enuresis), REM sleep-related problems, and morning sleep inertia. Children with FOH often experience thermal discomfort (e.g., feeling hot, excessive sweating) in neutral ambient temperature conditions, as well as no discomfort during exposure to the extreme cold, and alternate noticeably between being excessively hot in the evening and cold in the morning. We hypothesized that these sleep- and temperature-related symptoms were overt symptoms of an impaired ability to dissipate heat, particularly in the evening hours near the time of sleep onset. We measured sleep/wake variables using actigraphy, and nocturnal skin temperature variables using thermal patches and a wireless device, and compared these data between children with PBD/FOH and a control sample of healthy children. The results are suggestive of a thermoregulatory dysfunction that is associated with sleep onset difficulties. Further, they are consistent with our hypothesis that alterations in neural circuitry common to thermoregulation and emotion regulation underlie affective and behavioral symptoms of the FOH phenotype.

## 1. Introduction

Sleep disturbance is a common feature of mood disorders. In children diagnosed with pediatric bipolar disorder (PBD), problems with the quality of both sleep and wakefulness are prominent, and include bedtime refusal, sleep onset insomnia, parasomnias, morning sleep inertia, and daily bouts of both hyperactivity and hypoactivity [1,2,3,4,5,6,7,8,9,10,11,12,13,14]. An associated observation is that children with BD alternate noticeably between being excessively hot in the evening and cold in the morning [15,16]. Despite the pervasiveness of parental complaints and frequent clinical observations of sleep-related issues, there are few studies of sleep and rest/activity patterns in PBD, and data relating to temperature regulation in PBD are virtually non-existent. It is likely that methodological complexities of obtaining these data in children with psychiatric disorders are a primary reason for the lack of such studies. In addition, the very concept of childhood-onset bipolar disorder has been controversial, and only in the last decade have empirical investigations begun to characterize symptoms and examine potential etiologies of bipolar mood disorder in children [17,18]. 

A majority of children with bipolar disorder exhibit a subsyndromal course of illness. This has prompted many investigative groups to explore whether such a presentation is developmental or unique. Despite the ongoing debate, there has been a rapid increase in the rate of diagnoses of bipolar disorder in children (e.g., [19]). Concurrently, breakthroughs in neurology, neuroimaging, and genetics have called into question the existing conceptually-based psychiatric constructs altogether. New dimensional* versus* previous categorical research approaches which reflect these advances have made progress toward identifying dimensions of symptoms, or phenotypes, that are more likely to lead to evidence-based diagnosis and treatment. Such an example is a novel phenotype of PBD called Fear of Harm (FOH) [20,21,22]. 

The FOH phenotype includes symptoms that have not previously been associated with a nosological definition of bipolar disorder or other proposed childhood phenotypes of PBD [22]. However, many of these symptoms are recognized as co-morbid with the condition. In particular, separation anxiety, sleep/arousal disorders, parasomnias (night-terrors, enuresis) and REM sleep-related problems are primary features of the FOH phenotype. In addition, children with FOH often experience thermal discomfort (e.g., feeling hot, excessive sweating) in neutral ambient temperature conditions, as well as no discomfort during exposure to the extreme cold [23]. It is conceivable that an environmental signal expected to promote cold-defensive responses and lead an individual to seek warmth, to escape the cold, and to stimulate thermogenesis is not registered or not responded to, or the sensation/perception of change in cold ambient temperature is muted in these children, and therefore there is no aversive response generated by central thermoregulatory mechanisms.

An in-depth analysis of a large sample of children at risk for, or with a community diagnosis of, bipolar disorder, indicated that the population divides into approximate thirds of no-FOH, low-FOH, and high-FOH [22]. Compared with children in the no or low FOH groups, children with high FOH have significantly higher indices of severity of mania and depression [22,23], and clearly fall within the domain of classical manic depression. Course of illness analysis has indicated that presence of the FOH trait associates with the most severe form of the illness, including early age of onset, frequent hospitalizations, significant social impairment, and school problems [22].

Children with the FOH phenotype, and bipolar disorder in general, also often experience sleep onset insomnia symptoms [1,6,7,8,9,11,12,13] that are typically reported in younger children as “bedtime refusal” or “difficulty settling at night”. Older children self-report an inability to fall asleep, sometimes concurrent with racing thoughts and psychomotor agitation. In addition, both children and their parents complain of the children’s severe morning lethargy and an inability to awaken spontaneously until later than similarly aged healthy children. We have hypothesized that these sleep- and temperature-related symptoms may be the overt symptoms of an impaired ability to dissipate heat, particularly in the evening hours near the time of sleep onset.

In an initial effort to confirm parental and clinical observations of these sleep and temperature problems, we examined rest/activity patterns using actigraphy, sleep parameters using parent-completed Sleep Diaries, and nocturnal skin temperature variables using a wireless temperature monitoring device, in children diagnosed with bipolar disorder, who met criteria for the FOH phenotype. These data were compared between children with FOH with a control sample of healthy children. With this approach we attempted to obtain high-quality objective data in a naturalistic setting, while circumventing some of the practical difficulties of studying sleep and temperature in children. The actigraphy and Sleep Diary data were utilized to obtain quantitative and qualitative information about sleep. Skin temperature data were utilized to examine relationships between a proxy measure of heat dissipation (*i.e.*, the distal-proximal gradient) and its relationship to sleep parameters, particularly latency to sleep onset. 

We hypothesized that relative to the controls, children with PBD/FOH would have difficulty dissipating heat at bedtime, and further that this thermoregulatory symptom would be associated with longer latencies to sleep.

## 2. Experimental Section

### 2.1. Subjects

The study was reviewed and approved by the Weill Cornell Committee on Human Subjects in Research (Weill Cornell IRB). Potential subjects were recruited primarily via the research studies portal on the website of the Juvenile Bipolar Research Foundation. The aim of this initial screening methodology was to contact parents/caregivers of children who met criteria for the FOH phenotype, as described in detail in Papolos* et al.* (2009) [23], and controls with no psychiatric symptoms or history. Parents or caregivers completed the online Child Bipolar Questionnaire (CBQ [23]) and consented to being contacted if the responses indicated initial eligibility for the research study. Eligible subjects met the following criteria: CBQ total score >65, plus endorsed as being present almost always or always at least three of five sleep-related items on the CBQ, plus the CBQ item relating to rapid, abrupt mood swings, plus the CBQ item relating to thermal discomfort. Additional eligibility criteria (e.g., age 5–12 years old) were assessed via contact from the subject recruiter to the parent/caregiver respondent, and the study protocol was described. Eligible and interested adult/child teams were mailed study consent/assent forms. (Separate versions of assent forms were provided for children 5–7 years old* versus* 8–12 years old, as stipulated by the Weill Cornell IRB). 

Following consent/assent, a diagnostic interview via telephone was conducted by a trained clinical rater using the K-SADS [24]. Diagnoses of DSM-IV Bipolar Disorder, or of no DSM-IV diagnoses for control subjects, were confirmed by expert consensus using information from the diagnostic interview and K-SADS. 

Reported here are data from 16 children with PBD/FOH (9M, 7F; mean age = 8 ± 2 years, range 5–12 years). Mean age of onset of PBD was 4.25 years. All met criteria for FOH phenotype [19]. Control data are from 4 subjects (4M, mean age = 8 ± 1 years, range 7–9 years). 

A study kit was provided to each adult/child subject team, and returned at the end of the protocol. The kit included a programmed Actiwatch (Respironics Minimitter, Inc., Bend, OR, USA), a programmed Vitalsense monitor with activated wireless dermal temperature patches (Respironics Minimitter, Bend, OR, USA), labeled Salivette tubes (Alpco Diagnostics, Inc., Wyndham, NH, USA) for saliva collection, and an instruction binder with 14 copies of a Sleep/Medication/Activity Diary (Diary). 

### 2.2. Protocol

#### 2.2.1. Diary

On each one-page Diary the adult/child team cooperated to complete questions with quantitative answers about the time the child got into bed the previous night, or started to fall asleep if not in bed (diary Bedtime), estimated Sleep Onset Latency (SOL), estimated Waketime (*i.e.*, awakening from sleep; WT), and time the child got out of bed in the morning (Risetime). Additional qualitative questions completed each morning included whether the child had experienced any long awakenings, nightmares, night terrors, or other parasomnias, got out of bed for any reason, or changed sleeping locations at any time during the night. The second portion of the Diary was completed each evening by the adult and included questions about any medications the child had taken during the day, with their administration time and dose, as well as questions to help adjudicate the Actiwatch data (e.g., periods of removal of the Actiwatch during the day, nap times). Adults/children were instructed to complete the portion of the Diary with questions about the previous night’s sleep period in the morning within 2 h of the child’s Risetime, and the portion with questions about the day’s activities and medication regimen in the evening hours. 

#### 2.2.2. Nocturnal Skin Temperature

The dermal patches were to be placed on the child’s (a) lower left calf (distal), and (b) subclavicle region (proximal) at least 1 h prior to anticipated bedtime on 3 nights to record overnight temperature. In order for the successful detection of the signal from dermal patches, the Vitalsense monitor needed to be within approximately 3 feet of each of the patches. While awake and out of bed, the child wore the monitor in a nylon waistpack. At bedtime, the monitor was placed near the child on a nightstand or in the bed next to the child. Although an attempt was made to record temperature for 3 consecutive nights and the intervening days, logistical and comfort considerations resulted in revised instructions for the adult to remove the sensors in the morning 1 h after the child got out of bed. The dermal patches transmitted skin temperature (*T*sk) to the Vitalsense monitor at a 1-min sampling rate and transmitted ambient temperature readings when the patches were removed from the child’s skin. Skin temperature readings from the time of placement on the skin until 4 h later (*i.e.*, incorporating bedtime and nocturnal sleep onset) were analyzed to obtain the absolute distal temperature, absolute proximal temperature, and distal-to-proximal gradient (DPG). 

#### 2.2.3. Actigraphy

A small wrist-worn Actiwatch-L (Respironics Minimitter, Bend, OR, USA) was programmed to obtain activity data at a 2-min sampling rate for up to 30 days. The child was to wear the Actiwatch continuously, except for periods when the water-resistant watch would remain submerged in water for an extended time (e.g., bathing, swimming). 

#### 2.2.4. Data Analysis

Diary data were summarized for each child individually and then by group (Control, FOH). Parameters included average BT, SOL, Time Spent Asleep, WT, RT, Sleep Period Duration from Bedtime to Risetime, and Sleep Efficiency (Time Spent Asleep/Sleep Period Duration). From the continuous Actiwatch data individual sleep periods were extracted for analysis, each constrained by the Diary-reported Bedtime and Risetime of the corresponding night. Each sleep period was analyzed via Actiware 5.0, applying a medium sensitivity algorithm to distinguish sleep from wake epochs, and to thus estimate actigraphy-derived Sleep Onset Latency (minutes from the clock time of Bedtime to 5 contiguous sleep epochs) and Sleep Efficiency (SE; # of sleep epochs during sleep period divided by total # of epochs during the sleep period).

#### 2.2.5. DPG/DPG0

Usable temperature data was operationally defined as data from nights in which *T*sk from both distal and proximal sensors was recorded starting a minimum of 30 min prior to Bedtime and continued for at least 3 h after Bedtime. The proximal-minus-distal *T*sk difference was calculated for each minute for each night. The resulting curve was the DPG curve. DPG0° was defined for each night as the clock time at which the DPG curve first crossed 0°. The difference, or gradient, between proximal and distal skin temperature has been validated as a measure of heat dissipation, a thermoregulatory process in which heat is shunted from the body’s core to its shell [25,26,27,28,29]. The distal-proximal gradient (DPG) has a temporal relationship to sleep onset in normal subjects, such that when the gradient approaches 0°, sleep onset is imminent [25,26,27,30,31,32]. This temporal relationship has been shown to be more than coincident; peripheral heat loss through skin of the extremities appears to be functionally related to, and permissive of sleep onset [25,26,31,32]. In populations with compromised thermoregulatory function, this temporal relationship has been shown to be disrupted, with sleep onset difficulties as one result [28,32,33,34,35]. Converging evidence for the functional role of heat dissipation in sleep onset indicates that promoting heat dissipation in those with sleep onset difficulties facilitates sleep induction [26,29,34].

Sleep, actigraphy, and temperature parameters were compared between Control and FOH groups using *t*-tests, or Mann Whitney *U* tests. While the unit of analysis was a parameter from an individual night, degrees of freedom (when applicable) were based on the number of subjects from each group contributing to the analysis. Spearman rank-order correlations estimated the strength of relationships between variables, including between ordinal and continuous variables.

## 3. Results and Discussion

From the 16 FOH children, usable Diary and Actiwatch data were obtained from 92 nights* versus* 37 nights from the 4 Control children. Coincident, usable Diary, Actiwatch, and temperature data were obtained from 26 nights from 10 children in FOH* versus* 8 nights from 4 children in Control. 

### 3.1. Sleep

Table 1 shows results of sleep-related parameters calculated from the Diary and Actiwatch. Neither Bedtime nor Risetime differed significantly between FOH and Control children, and thus sleep period duration did not differ between groups. However, sleep onset latency from both Diary and actigraphy was 2–3 times longer in FOH than Control groups. As calculated from Actiwatch data, SE did not differ between groups, but was relatively low for both groups, which may reflect (a) the sensitivity of the algorithm applied to determine wakefulness* versus* sleep (medium sensitivity was used); (b) (related to the algorithm sensitivity) the detection of restlessness during sleep in both groups of children; and/or (c) relatively poor sleep, on average, in both FOH and Control children. A recalculation of sleep parameters from Actiwatch data using a less sensitive algorithm (*i.e.*, higher threshold for determining wakefulness) increased the SE ratio equivalently in both groups. 

**Table 1:** Sleep parameters in Controls and Children with Fear of Harm phenotype.

Sleep Parameter (Source)	Control	FOH
Bedtime (d)	21:04 ± 0:40	21:10 ± 1:01
Risetime (d)	06:32 ± 0:50	07:23 ± 1:04
Sleep Onset Latency (d)	9 ± 5 min	27 ± 20 min
Sleep Onset Latency (a)	8 ± 4 min	37 ± 38 min
Total Sleep Time (a)	7 h 36 min ± 41 min	8 h 07 min ± 1 h 17 min
Sleep Efficiency * (a)	85.7% ± 7.1%	87.0% ± 6.2%
Sleep Period Duration (d)	9 h 13 min ± 44 min	10 h 04 min ± 1 h 28 min
Parasomnias reported	0	8

Source: (a) = derived from actigraphy; (d) = from Diary. * Sleep Efficiency is the ratio of Total Sleep Time to the interval from Sleep Onset to Risetime.

### 3.2. Parasomnias

In the control group, there was not a single report of a parasomnia event on the Diary. In the FOH group, Diaries from 8/16 subjects indicated that the child had experienced at least one parasomnia event on a given night. More than one unique parasomnia and more than one parasomnia event on multiple nights were reported in all but one of these eight children. The parasomnias reported were primarily nightmares, reported from six children, with two episodes of enuresis from the same child and three episodes of night terrors from three different children. 

### 3.3. Skin Temperature and Sleep

The protocol indicated that skin temperature recording via the dermal patches and the Vitalsense monitor should begin at least 60 min prior to anticipated bedtime, or 1800 h, whichever was earlier. However, the average start time of skin temperature recording was 2012 h. This was, on a majority of nights, at least an hour prior to Bedtime. 

**Figure 1:**
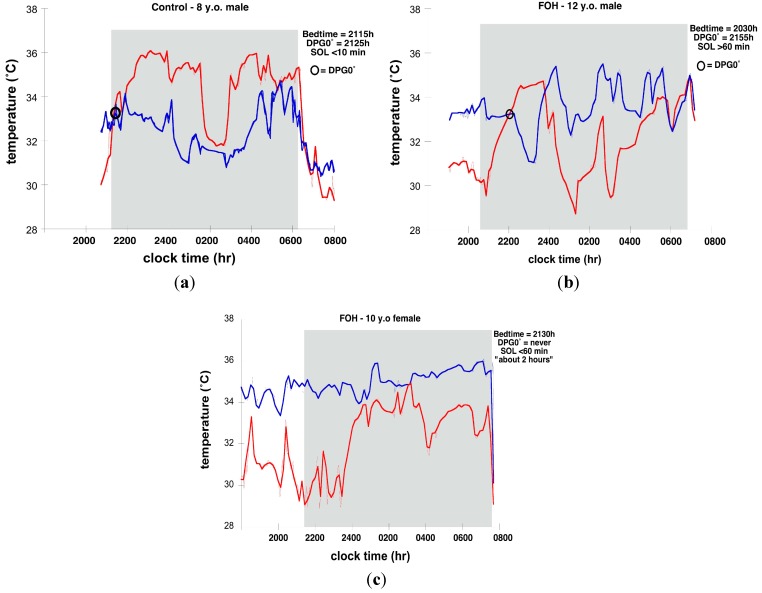
Skin temperature and sleep timing in three children: (**a**) 8-Year old male Control (red line = distal temperature; blue line = proximal temperature); (**b**) 12-Year old male Fear of Harm (FOH) (red line = distal temperature; blue line = proximal temperature); (**c**) 10-Year old female FOH (red line = distal temperature; blue line = proximal temperature).

**Figure 2:**
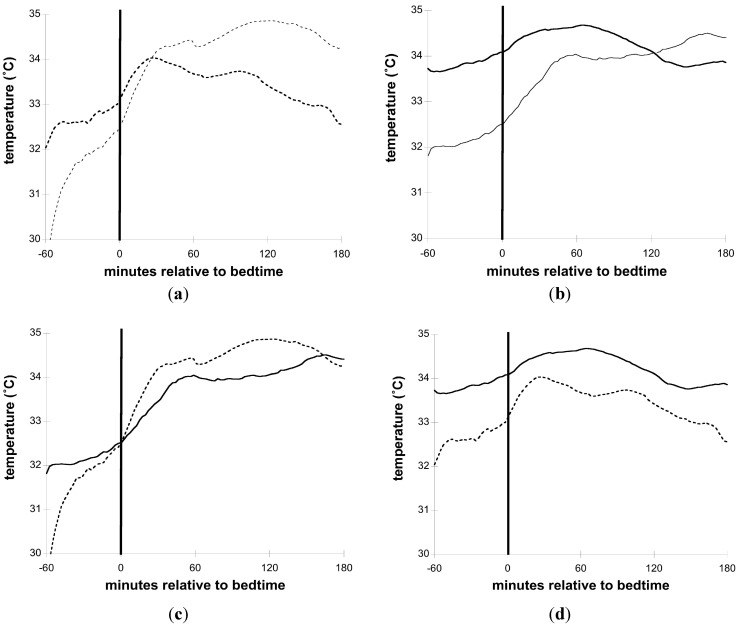
Skin temperature relative to Bedtime. Smoothed curves from group averages of distal and proximal temperature from the 60 min before until 180 min after Bedtime; (**a**) Control (thin dotted line = distal; thick dotted line = proximal); (**b**) FOH (thin solid line = distal; thick solid line = proximal); (**c**) Control *vs*. FOH distal (dotted line = Control; solid line = FOH); (**d**) Control *vs.* FOH Proximal (dotted line = Control; solid line = FOH).

Figure 1a–c illustrates distal and proximal *T*sk and sleep timing on individual nights from 2 FOH and 1 Control. Figure 2a–d illustrate group mean *T*sk curves for the interval from 1 h (−60 min) before until 3 h (+180 min) after Bedtime. 

The average absolute distal *T*sk levels for the −45 min to +180 min interval surrounding Bedtime for all nights for Control and FOH groups did not differ. In particular, at Bedtime, the absolute distal *T*sk levels were essentially identical for both groups (control: 32.30°, FOH: 32.33°). However, proximal *T*sk averaged more than 1.2 °C higher in the FOH relative to Control group across the same interval. The largest magnitude of difference in absolute proximal *T*sk levels occurred in the interval from 45 min prior to Bedtime through 15 min after Bedtime. In the Control group, proximal *T*sk initially increased slightly at Bedtime, but as distal *T*sk increased substantially and quickly after Bedtime, proximal *T*sk started slowly decreasing, resulting in the DPG0° an average of 9 min after Bedtime. 

In FOH children, a different pattern of Tsk around Bedtime was observed. On 4 nights from three different children in the FOH group, a DPG0° did not occur in the −60 min to +180 min analysis interval. While distal Tsk increased around Bedtime in a manner similar to Controls, proximal Tsk remained higher before and in the 3 h after Bedtime in FOH children. 

Due primarily to the lag in proximal Tsk decrease, the time at which DPG0° occurred was significantly later in FOH children. Analyses of Tsk relative to sleep revealed that DPG0° averaged 2114 h ± 40 min for Control and 2212 h ± 1 h 16 min for FOH groups, respectively (*t* = 2.87, *p* < 0.05). Similarly, there was a delay in the DPG0° relative to Bedtime in the FOH group, even though Bedtime did not differ between the groups. The interval from Bedtime to DPG0° averaged 11 min ± 15 min for Control children compared with 61 min ± 51 min for FOH children (*t* = 3.21, *p* < 0.01). 

**Figure 3:**
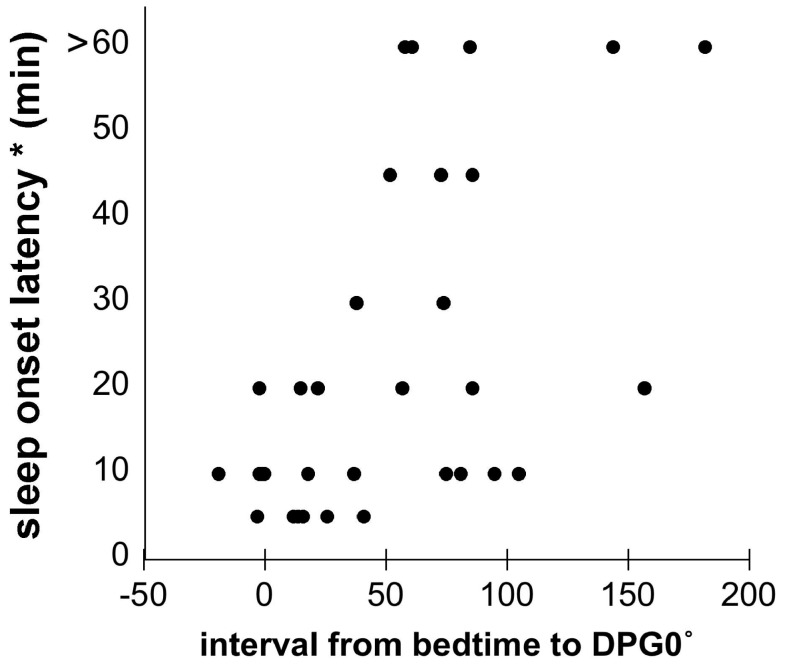
Relationship between interval from Bedtime to distal-proximal gradient of 0 degrees and sleep onset latency for individual nights with coincident Diary and skin temperature data from both Control and FOH subjects (Spearman’s rho = 0.48; *p* < 0.05).

This lag between Bedtime and DPG0° was associated with a longer latency to sleep onset. SOL estimated by the Diary correlated significantly with the interval from Bedtime to DPG0° (SOL/BT-to-DPG0° interval: Spearman’s rho = 0.48, *p* < 0.05; Figure 3). There was a similar trend for actigraphy-derived SOL (actigraphy-derived SOL/BT-to-DPG0° interval: Spearman’s rho = 0.36, *p* = 0.12).

It was the common theme of parental and clinical reports that children with FOH exhibit signs and symptoms of altered thermoregulation that led to the current study. For example, children diagnosed with BD often have unusually reddened cheeks and ears, wear few layers of clothes in cold temperatures, and frequently complain of being hot even when others are comfortable. The anecdotal evidence for this phenomenon is quite widespread, and evidence for disturbed temperature regulation, particularly in the circadian domain, has been previously described in adults with affective illness [25,26,27], but to our knowledge, no systematic investigations of temperature perception or thermoregulation in PBD have been conducted.

An intimate association between sleep and body temperature has long been recognized, but a renewed interest in research on this topic has revealed that temperature regulation influences sleep to a greater degree than previously known. A growing body of evidence indicates that declines in core temperature and increases in peripheral heat loss may be functionally related to sleep initiation and consolidation [28,29,30,31,32,33,34,35]. We and others have found that when the maximum rate of decline in core temperature (*i.e.*, the steepest slope) occurs prior, and in close proximity to bedtime, sleep onset latency is reduced, and slow wave sleep is increased [28,31]. An elegant series of studies by Krauchi and colleagues determined that peripheral heat loss via distal vasodilation, which drives the nocturnal decline in core temperature, is a permissive condition for sleep initiation [28,29,30]. In brief, they find that sleep onset occurs when the DPG approaches 0 degrees. The DPG0° was a better predictor of sleep onset than core body temperature, its rate of change, heart rate change, melatonin levels, or subjective ratings of sleepiness [28]. A complementary body of work by van Someren and colleagues has systematically demonstrated the role of skin temperature in sleepiness (e.g., [32,33]), and how manipulating the amount of heat dissipated via the skin can alter centrally-regulated vigilance levels and sleep propensity [34,35].

Some types of insomnia are associated with heat dissipation problems. Individuals with vasospastic syndrome, who have deficient vasodilation capacity, require twice as long to fall asleep as healthy controls [36]. Also, compromised capacity to lose heat from the periphery has been hypothesized to largely account for sleep maintenance insomnia in elderly individuals [37,38,39] and in women with menopausal hot flashes or night sweats during sleep [40]. It is conceivable that children with BD have thermoregulatory dysfunction that affects the capacity for heat dissipation and thereby interferes with the sleep initiation process. It is further possible that the neural mechanisms underlying the disruption in both thermoregulation and sleep regulation also modulate or mediate emotion dysregulation in these children.

## 4. Conclusions

The current data add to the emerging evidence for physiological and behavioral underpinnings of the FOH phenotype of pediatric bipolar disorder. Objective evidence of sleep disturbance, in the form of long sleep onset latencies, is in agreement with a large body of anecdotal, questionnaire, and empirical evidence for sleep problems in these children. Although neither Bedtime/Risetime, nor sleep period duration were obviously aberrant in the FOH children in this study, the average Diary-estimated sleep onset latency of greater than 30 min is comparable to that of sleep onset insomniacs. 

It is necessary to note the numerous and varied difficulties with this in-home study, which limit interpretation of these data. As one result of these mostly logistical difficulties obtaining reliable diary, skin temperature, and sleep/wake data in children, the number of usable datasets (defined as having temperature on at least one night, and Actiwatch data for at least 7 nights) obtained were from a far smaller number of subjects than were enrolled in the study. Nonetheless, the clinical characteristics of these subjects (and Controls) are well-defined, the sample is well-characterized, and the information from the small sample is compelling. 

The correlational nature of these results and the small sample size proscribe attributing directionality of effects among temperature, sleep, and emotion regulation disturbances. Nonetheless, the results are suggestive of a thermoregulatory dysfunction that is associated with sleep onset difficulties in children with a clear dysregulation of emotion as manifest in the Fear of Harm phenotype. They are consistent with our hypothesis that alterations in neural circuitry common to thermoregulation and emotion regulation, involving the orexin system, underlie affective and behavioral symptoms of the FOH phenotype [22]. At a minimum, additional studies of thermoregulation in children with this psychiatric condition are warranted.
